# Mini review: human clinical studies of stem cell therapy in keratoconus

**DOI:** 10.1186/s12886-024-03297-w

**Published:** 2024-01-23

**Authors:** Masoumeh Ahadi, Shahrokh Ramin, Ali Abbasi, Hanieh Tahmouri, Seyed Bagher Hosseini

**Affiliations:** 1 Department of Optometry, School of Rehabilitation Sciences, Shahid Beheshti University of Medical Sciences, Tehran, Iran; 2 Department of Optometry, School of Rehabilitation Sciences, Incubation Center for Pharmaceutical Technology (ICPT), Cell Therapy Department, Red Crescent Pharmaceutical and Clinical Complex, Shahid Beheshti University of Medical Sciences, Tehran, Iran; 3 Department of Ophthalmology, Eye Bank of Islamic Republic of Iran, Shahid Beheshti University of Medical Sciences, Tehran, Iran

**Keywords:** Keratocytes, Regenerative Medicine, Keratoconus, Stem Cell Therapy, Mesenchymal Stem Cells

## Abstract

Treatment of keratoconus is one of the most interesting research fields for researchers in the world. Regenerative medicine based on human stem cells in the treatment of keratoconus has recently received attention. Despite extensive laboratory and animal studies in regenerative medicine of cornea, there are limited clinical studies in keratoconus. These studies showed promising results of stem cell therapy. In initial studies, the transplantation of these cells into stroma was associated with increased vision and improved corneal parameters without side effects. In this article, we tried to review different aspects of keratoconus stem cell therapy, including cell extraction and culture, surgical procedure, effectiveness and safety of this method in human clinical studies.

## Background

The cornea consists of 5 different layers, 90% of its thickness is related to the middle part or the corneal stroma. Corneal stroma is composed of collagen lamellae, extracellular matrix components, and keratocytes. Keratocytes are specialized fibroblasts located between collagen lamellae. Keratocytes are quiescent cells, but they actively synthesize the extracellular matrix (ECM). Keratocytes play a vital role in maintaining the transparency of the cornea, healing its wounds, and producing its components. In stromal injuries, these cells undergo apoptosis or are replaced by other cells [1–3]. One of the disorders of the corneal stroma is keratoconus, which the thinning and steepening of the cornea leads to irregular astigmatism and blurred vision. Although the main cause of keratoconus is not known, it is related to several risk factors such as family history, age, race, certain disorders, inflammation, and eye rubbing. Thinning of the stroma occurs due to the decrease in the number of lamellae and destruction of collagen by proteolytic enzymes, cytokines and free radicals and abnormal keratocytes [4, 5]. Studies on keratoconus patients have demonstrated decreased keratocyte density in the anterior and posterior stroma due to abnormal function of the keratocytes and the high rates of cell death and loss of ECM. Keratocyte loss is not related to corneal thickness and disease stage. In addition, a decrease in the density of keratocytes in the anterior and posterior stroma of keratoconus patients who wear contact lenses has been reported [6–10].

Depending on the stage of keratoconus and the severity of vision loss, there are various optical and surgical treatments. The basics of treatment are based on increasing the strength of the cornea, reducing the irregularities of the cornea and improving the refractive parameters, which often provide good vision for patients. Despite many advances in treatment, the visual results in some cases, such as corneal thinning and corneal scarring, are not satisfactory, and researchers are looking for new methods [10–13]. Since keratoconus is associated with decreased keratocytes, researchers considered cellular therapy as a novel technique to replace and increase the number of keratocytes and improve keratocyte function. Cellular therapy is performed with various methods such as tissue engineering and stem cell therapy. In animal models, intrastromal injection of human keratocytes led to a re-increase in the number of stromal cells and improved their function. However, propagation keratocytes in vitro, need to many growth factors for cell culture, obtaining a large number of these cell for tissue engineering, and the immune system response to new cells are challenging [14–17]. In experimental animal studies, human-derived mesenchymal stem cells were not only able to differentiate into keratocytes, but also survived inside the cornea and showed the same function as host stromal cells [18–21]. Along with animal studies, clinical studies have been conducted intrastromal transplantation of stem cell and showed promising results. The first study was performed by Alio and colleagues in 2017 and then followed by other researchers [22, 23].

In this article, we reviewed human clinical studies of stem cell therapy in keratoconus. Due to the novelty of this treatment method, there were some articles in which we present the reported contents and results of these studies.

## Materials and Methods

### Stem cell sources used in clinical studies

In terms of origin, stem cells are divided into two major categories: embryonic cells and adult stem cells. Adult stem cells are usually used in clinical studies. These cells are found throughout the body of children and adults. One of the most important adult stem cells is mesenchymal cells, which participate in regeneration of tissues of mesenchymal origin. Mesenchymal cells have the ability to regenerate themselves and transform into different cell lines. The ease of isolation and autologous nature of mesenchymal cells has made them suitable candidates for cell therapy [24–27]. Human mesenchymal cells are obtained from adipose tissue, bone marrow, umbilical cord, dental pulp, gum, hair follicle, cornea, and fetus. Although corneal stem cells seem ideal for cell therapy, it is difficult to obtain a sufficient amount of cells, and it must be done from another healthy eye, which is not always possible. In the early years, bone marrow was the most important source of mesenchymal stem cells in tissue engineering, but recently adipose-derived stem cells has attracted a lot of attention due to its large amount of stem cells compared to bone marrow and its ability to be transplanted into the host. Also, it is easier to isolate these cells from adipose tissue than bone marrow with minimal risk. Compared to bone marrow stromal cells, adipose tissue stem cells are more resistant in laboratory culture and have a higher proliferation rate. Therefore, human adipose tissue is a suitable source of extraocular stem cells due to its easy access, proper efficiency in cell recovery, and high ability to differentiate into all types of cells such as keratocytes [18, 28–32].

### Isolation, proliferation and identification of adipose derived stem cells

The standard liposuction method is usually used to isolate adipose-derived stem cells (ASCs). After local anesthesia, 20–30 ml of adipose tissue samples are taken from the abdomen and sides by a specialist, under sterile conditions. The adipose tissue sample is washed in phosphate-buffered saline (PBS) and placed in collagenase I solution at 37° C and then collagenase activity is inhibited by adding autologous human serum10% (AHS). Erythrocytes are lysed and removed by erythrocyte lubrication buffer. Then the sediment cells are cultured in the Dulbecco’s Modified Eagle Medium (DMEM), including glutamex and sodium pyrotate, 10% autochemal serum, 1% penicillin-sterropetomycin, amphotericin 2%. After three cell passages, the phenotype of cultured cells is evaluated by flow cytometry. Mesenchymal cells are characterized by CD45, CD235a, CD31, CD34 + markers. Also, other markers according to cell surface antigens are: CD13, CD73, CD90 and CD105. Mesenchymal cells in vitro show a stable phenotype and remain in a single layer and are prepared to differentiate into special cells. 60 to 80 h before surgery to resemble the physiological condition of the stromal keratocytes, ASCs are rested by reducing autologous human serum concentration to 0.5%. The stem cells are prepared to be injected into the corneal stroma [23, 33].

### Surgical techniques for stem cells implantation

Implantation of stem cells with femtosecond laser is done with the following two methods:

### Intrastromal implantation of stem cells alone

The procedure is performed under topical anesthesia in the operating room. After marking the corneal center, using the femtosecond laser, a lamellar incision is created in the corneal stroma with a diameter of 7.5 -8.0 mm, and the location of this incision based on pachymetry findings in the half-depth of the cornea. The femtosecond laser parameter settings are similar to implantation of intrastromal ring. A 30 degrees anterior side cut incision was made and then, the intrastromal pocket was opened with a Morlet lamellar dissector. Then, 1 cc of the solution containing 3 × 10^6^ ASCs was injected into the stromal pocket. In fact, what is done is the irrigation of the stromal pocket with the solution containing ASCs, and as expected, more fluids will be removed and few cells remain and differentiate. Due to the small size of the injection site, there is no need to perform sutures. At the end, a bandage lens was placed to improve the corneal incision for a week. Postoperatively, patients were given combined topical antibiotics and steroids for 2 to 3 weeks depending on the degree of recovery [34–36].

### Intrastromal implantation of stem cells with a decellularized stromal scaffold

Immunological rejection is one of the potential side effects of allogeneic corneal transplantation in keratoconus patients, especially in repeated transplants and corneal scarring or vascularized cornea. The use of non-immunogenic corneas would be effective in these patients. These corneas are allogeneic decellularized corneal scaffolds which cells and genetic material have been removed in the laboratory. Additionally, decellularized corneal scaffolds together with autogenic keratocytes provide a more natural environment for cell growth and differentiation and are well tolerated by hosts. There are several methods to decellularize corneal tissue using physical or chemical means. Protocol of preparation decellularized stromal scaffold is based on the study of Alio et al [37–39]. The origin of corneal stromal scaffolds is the donated corneas available in the eye bank. First, the corneal epithelium is mechanically removed, then a corneal lamina 120 μm thick and 9.0 mm in diameter is cut by a femtosecond laser. Laser settings are used according to the parameters of Lasik surgery. Corneal lamina is obtained from the corneal stroma, which does not differ from its anterior or posterior parts. The corneal lamina is washed in PBS containing 1% antibiotic-antimicotic. The laminas is placed in 1% sodium dodecyl sulfate solution with a protease inhibitor cocktail. The lamina is subsequently washed in PBS with 1% antibiotic-antimicotic. Then, the lamina is incubated in DNAse in PBS with the same protease inhibitor cocktail. After DNA removal, the lamina is washed in PBS with 1% antibiotic-antimicotic. 24 h before surgery, recellularization of corneal scaffolds is performed by the culture of autologous ASCs (1 × 10^6^/1 mL) of the patient on the lamina. Now, the recellularized lamina is ready to implant.

Surgery is performed under topical anesthesia with oral sedation. Like the previous method, a stromal pocket is created into the stroma by using femtosecond laser. After opening the stromal pocket, the corneal lamina is placed inside. Also, the inside of stromal pocket is washed with a solution containing an additional 1 × 10^6^ autologous ASCs in 1 mL PBS before and after the lamina implantation. To prevent the increase of intraocular pressure, a temporal limbal paracentesis of the aqueous humor is performed before placing the lamina. At the end, the surgical site is sutured with one interrupted 10/0 Nylon suture, which are removed one week later. Finally, Topical antibiotic and steroids is prescribed. This method is related to the publication of Alio et al., which is described in detail [35, 38].

### Clinical studies

Two groups have conducted research in this field, the results of which are stated here. Table 1 shows a summary of the results of these clinical studies.

**Table 1:** Summary of clinical studies of stem cell therapy on keratoconus

Author(Year)	Participant characteristics	Conclusions
Alio et al. (2021) [23, 39]	Intrastromal implantation of stem cells alone: 5 eyes of 5 patients with advanced keratoconus, F/U: 3 years	Side effects: no evidences in examination
		BCVA: 0.18 lines in Log MAR scale
		Sph refraction: 1.1D improvement
		Cyl refraction: 0.50D improvement
		Max keratometry: 2.0 D improvement
		CCT: 30 μm increase
		Aberrometry findings: significantly reduced
		Density of stromal keratocytes: increased
	Intrastromal implantation of decellularized stromal scaffold: 5 eyes of 5patients with advanced keratoconus, F/U:3 years	Side effects: no evidences in examination
		BCVA: 0.18 lines in Log MAR scale
		Sph refraction: 1.0D improvement
		Cyl refraction: 1.50D improvement
		Max. keratometry: 2.0 D improvement
		CCT: 31 μm increase
		Aberrometry findings: significantly reduced
	Intrastromal implantation of stem cells with a decellularized stromal scaffold: 4 eyes of 4 patients with advanced keratoconus, F/U:3 years	Side effects: no evidences in examination
		BCVA: 0.19 lines in Log MAR scale
		Sph refraction: 1.2D improvement
		Cyl refraction: 1.1D improvement
		Max. keratometry: 2.0 D improvement
		CCT: 30 μm increase
		Aberrometry findings: significantly reduced
		Density of stromal keratocytes: increased
Ramin et al. (2023) [36, 40]	Intrastromal implantation of stem cells alone:	Side effects: no evidences in examination
	8 eyes of 8 patients with moderate to severe keratoconus, age:22–35 years, F/U: 6 months	BCVA: 1.85 lines in Snellen scale
		Sph refraction: 0.34D improvement
		Cyl refraction: 0.84D improvement
		Ant. keratometry: 0.76D improvement
		CCT: 6.29 μm increase
		Aberrometry findings: reduced
		Density of stromal keratocytes: increased
	1 eye with severe keratoconus and central corneal scaring, age: 65 years, F/U: 6 months	Side effects: no evidences in examination
		BCVA: improvement of visual acuity from HM to 20/80 in Snellen scale
		Sph & Cyl refraction: marked improvement
		Keratometry: marked improvement
		CCT: marked improvement
		Aberrometry findings: marked reduced
		Density of stromal keratocytes: increased

*F/U* Follow-up, *BCVA* Best corrected visual acuity, *Sph refraction* Spherical refraction, *Cyl refraction* Cylindrical refraction, *CCT* Central corneal thickness, *Ant. Keratometry* Anterior mean keratometry

### Alio's research group and colleagues

As the first-in-human trial, Alio and colleagues studied the safety and efficacy implantation of human autologous ASCs in patients with advanced keratoconus in 2017. The patients of this study distributed into three groups. Implantation of autologous ASCs performed in 5 patients as group 1 (G1). In 5 patients in group 2 (G2), decellularized donor lamina was implanted and in 4 patients in group 3 (G3), recellularized donor lamina with autologous ASCs did. During 3-year assessments, no complications were observed. In the patients who had corneal lamina transplanted, in the first month after the operation, they had a mild early haziness of the implanted lamina, which improved. No patient had visual loss. Best corrected visual acuity (BCVA) of the patients in G1 increased 0.11, 0.20, and 0.18 in decimal lines mean value equivalents in Log Mar scale at 6, 12, and 36 months of follow up. BCVA of patients with decellularized (G2) or recellularized (G3) laminas improved 0.11, 0.2, 0.18 and 0.12, 0.2, 0.19 in Log Mar scale respectively after 6, 12, and 36 months. Central corneal thickness and corneal volume in all patients in G1 increased 36, 59, 30 μm, and 3, 3, 4 mm^3^ respectively during 6, 12, and 36 months of follow-up. The spherical refraction of the patients in G1 decreased of 0.8, 1.3, 1.1 diopters (D) at 6, 12, and 36 months. The cylindrical refraction of the patients remained almost stable during 12 months post-operative and decreased by 0.5 D after 36 months. The spherical refraction of the patients in G2 and G3 improved 1.0 D and 1.2D respectively in 36 months follow-up assessments and it was similar to results of G1 patients. The anterior mean keratometry of the patients in G1 decreased by 1 D and 2 D during 12 and 36 months respectively. The posterior mean keratometry in these patients remained stable in 36 months assessments. The maximum keratometry (Kmax) decreased by 1 D after 36 months. Also, keratometry findings in patients with decellularized and recellularized laminas showed results near to patients’ results with implantation of ASCs. In addition, aberrometry findings were improved in all patients during 3 years of follow-up. In confocal biomicroscopy findings, ASCs survival was confirmed and the number of keratocytes in all layers of stroma increased. The shape of the cells changed from round to fusiform after 6 and 12 months later, they were not distinguishable from regular keratocytes. OCT assessments demonstrated new collagen production at the surgical plane. No complications and no inflammatory responses were observed [23, 35, 39].

### Ramin's research group and colleagues

**Fig. 1:**
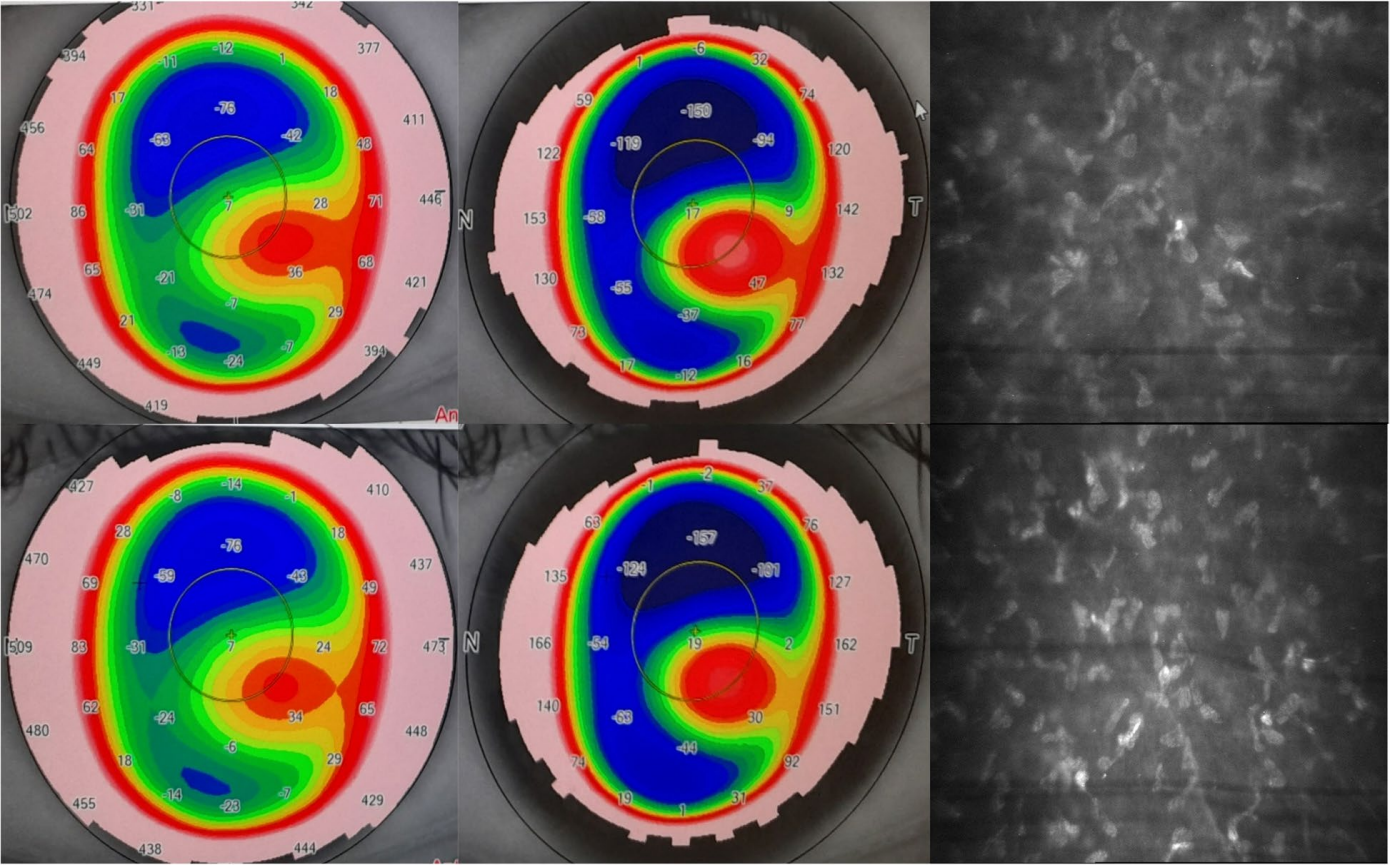
Ocular finding of the 32 years old after intrastromal injection of ASCs. Top: Preoperative data in the patient with BCVA of 0.3 with the mean equivalent refraction of -9.50D and the mean central corneal thickness of 423 μm. Bottom: 6 months postoperative data in the same patient with BCVA of 0.5 with mean equivalent refraction of -8.50D and the mean central corneal thickness of 432 μm. Left: The anterior keratometry of the patient, Middle: The posterior keratometry of the patient, Right: Corneal confocal biomicroscopy: in the anterior stroma ASCs (round and small cells) differentiated to new keratocytes (large, elongated and luminous cells)

In this study, Ramin et al. studied the safety and efficacy of intrastromal implantation of ASCs in keratoconus patients. They assessed 8 patients with moderate to severe keratoconus. In 6 months assessments, these patients showed no evidences of adverse effects such as corneal edema, scarring, and cell rejection. In confocal microscopy evaluations, the mean number of keratocytes of anterior and middle stroma was increased, but the mean number of keratocytes of posterior stroma was stable. The mean number of corneal endothelial cells did not differ after 6 months. The morphology of keratocytes and corneal endothelial cells was not different compared to the initial findings. These patients showed primary changes in first month and secondary changes after 3 months. The mean visual acuity of patients improved 1.85 lines with Snellen chart. The mean spherical refraction of patients improved 0.34 D and the mean cylindrical refraction of patients decreased 0.84D. While the anterior mean keratometry of patients decreased 0.76 D, the posterior mean keratometry remained stable (Fig. 1). The mean central corneal thickness and the mean corneal volume of patients increased 6.29 µm and 0.15 mm^3^ respectively. Aberrometry findings showed the reduction of corneal irregularities. Also spherical aberrometry and coma aberrometry decreased in patients. Additionally, one patient had no improvement of visual acuity and showed progressing of keratometry findings [36].

In another paper, this group implanted ASCs into stromal cornea in a patient with keratoconus and corneal scarring and a history of herpes simplex keratitis. The patient suffered from severe blurred vision (hand movement detection). Because very thin and scarified cornea, keratoplasty might be problematic. He underwent intrastromal injection of ASCs. At 6 follow-up assessments, corneal transparency increased and visual acuity improved to 20/80 with no adverse reactions. In topography finding, corneal irregularities significantly decreased and corneal thickness increased. ASCs implantation was safe and less invasive than the common corneal transplantation and can be considered as another possible option in corneal scarring [40].

## Discussion

Cell-based therapy, especially stem cells, shows promising results for the future of regenerative medicine in corneal diseases. One of the corneal diseases that can be treated with stem cells is keratoconus, the pathophysiology of which is related to the abnormalities of the corneal stroma and the loss of keratocytes. In in vitro and animal studies, stem cells not only differentiated into keratocytes but also promoted the function of host cells. In clinical studies, the use of stem cells has shown showed encouraging results [19, 20, 41–44]. In human studies, researchers transplanted autologous adipose derived stem cells by using femtosecond laser. The use of autologous stem cells means that there are no post-operative immunological problems. ASCs is easier to access adipose tissue cells and there is a large amount of them in fat tissue and these cells have more multi-lineage differentiation ability than other stem cells. Also, the use of femtosecond laser minimizes probable risks during surgery and subsequent problems  [22, 23, 36, 44].

In clinical studies, visual acuity of the patients improved between 0 and 4 lines during 6 to 36 month follow-up evaluations. The majority of patients experienced between 1 to 2 lines of visual improvement, and none had visual loss. This improvement in the patients' vision reflected changes in the thickness, curvature, and irregularities of the cornea that are referred to. In the patients with ASCs implantation, the spherical refraction decreased between 0.34 to 1.20 D and the cylindrical refraction improved 0.50D to 1.10D. In the patients with lamina implantation, refractive parameters improved 1.10 to 1.50D. While the anterior keratometry of the patients decreased between 0.84 to 1.0 D, the posterior keratometry of them remained stable. The central corneal thickness of the patients increased between 6.30 to 30.0 μm. The corneal aberrometry findings showed improvement of high order aberration and decreased irregularities. Density of keratocytes increased in stroma especially in its anterior and middle parts. New keratocytes were similar to host stromal cells and produced new collagen. Assessments of endothelium cells did not show any abnormality. The researchers did not report any side effects including edema, scarring and inflammation in the corneal layers in follow-up evaluations. Additionally, corneal transparency in a patient with severe corneal scaring improved significantly and visual acuity of the patient increased from hand motions detection to 20/80.

Since the reduction of keratocytes in the stroma is the prominent pathophysiology of keratoconus, the goal of intrastromal implantation of stem cells in keratoconus patients is to replace of keratocytes and increase their number. In fact, implantation of ASCs into stroma lead to primary and secondary changes. These changes are related to main and accompanying effects of stem cells. The primary changes are caused by the paracrine factors of stem cells, and the secondary changes are related to the differentiation of these cells into keratocytes [45–47]. In the early days after transplantation, stem cells release paracrine factors in the stroma, which promote host cell function. TNF-α-stimulated gene 6 protein (TSG-6) released by stem cells has the ability to inhibit the migration of neutrophils and reduce inflammatory processes in the cornea[45, 48, 49]. Also, stem cells secrete high level of hepatocyte growth factor (HGF) molecule, which prevents the transformation of fibroblasts into myofibroblasts and scar formation [46, 50]. The secretion of these factors improves the function of the patient's keratocytes by increasing the expression of stroma proteins and reviving the host's corneal keratocytes. Another effect of stem cell paracrine factors is to modulate immune responses in various ways. These cells reduce the penetration of inflammatory cells and CD68 + macrophages into stroma. Stem cells, on the one hand, reduce expression of the pre-inflammatory cytokine such as interleukin-2 (IL-2), IL17, monocyte chemoattractant protein-1 (MCP-1), interferon γ (IFNγ), macrophage inflammatory protein-1α (MIP-1α) and matrix metallopeptidase 2 (MMP2), and increase the expression of anti-inflammatory molecules IL-6, IL-10, TGFβ1, and TSG-6 becomes. Also, these cells reduce the expression of angiogenic factors including vascular endothelial growth factor (VEGF) and platelet-derived growth factor (PDGF) and increase the expression of anti-angiogenic intermediaries such as Pentraxin-3, and thrombospondin-1 (TSP-1) [14, 45, 47–50]. Modulation of immune responses by stem cells prevents the formation of fibrotic matrix components and remodulates stroma with normal structure and improvement of corneal transparency and reduction of corneal irregularities. The main effect of the treatment is related to the differentiation of stem cells into keratocytes, which occurs after 1–2 weeks and can be seen by changing the characteristics of stem cells to new keratocytes. Undifferentiated ASCs have rounded in shape and small diameter and after differentiation change to large and elongated keratocytes with a dendritic shape and luminous appearance. These cells produce and secrete extracellular matrix and stromal proteoglycans and help to regenerate the collagen matrix with a uniform diameter and regular inter-strand spacing similar to the host stroma [44, 50–53]. In general, the increase in the population of corneal keratocytes leads to stromal remodeling, reduced unfavorable refractive parameters of the cornea, and the improvement of the transparency of the cornea and the increase of visual acuity.

In conclusion, implantation of stem cells into stroma appears to be both safe and effective in keratoconus. In the initial studies, the transplantation of ASCs was associated with increased vision and improved corneal parameters and did not show any side effects. After transplantation, ASCs not only survive inside the stroma, but also differentiate into keratocytes and increase the number of keratocytes. New keratocytes produce and secrete collagen and proteoglycans and improve its structure. Also, paracrine factors of the ASCs and the immune modulating effects of the cells improve the performance of the host's keratocytes.

### Future research prospects

Despite the benefits of ASCs transplantation in keratoconus patients, this method is associated with challenges. Although it was effective in most patients, it had little effect in some of them. It seems that transplanting alone stem cells does not have enough power to make significant changes in corneal refractive parameters. Also, it is not known exactly how many cells injected into the stromal pocket will ultimately remain there to differentiate. In addition, the required amount of collagen renewal to regenerate thin and pathological corneas is not known. These and many other issues can be studied in the future. It is also suggested to use the results of this novel technique in keratoconus patients with thin cornea with and without corneal scarring in which standard methods are challenging.

## Data Availability

Not applicable.

## Abbreviations

ECM: Extracellular matrix
ASCs: Adipose-derived stem cells
PBS: Phosphate-buffered saline
AHS: Autologous human serum
DMEM: Dulbecco’s Modified Eagle Medium
BCVA: Best corrected visual acuity
Kmax: Maximum keratometry
MCP-1: Monocyte chemoattractant protein-1
IFNγ: Interferon γ
MIP-1α: Macrophage inflammatory protein-1α
MMP2: Matrix metallopeptidase 2
VEGF: Vascular endothelial growth factor
PDGF: Platelet-derived growth factor
TSP-1: Thrombospondin-1
